# Risk Factors for Incident Myopia among Teenaged Students of the Experimental Class of the Air Force in China

**DOI:** 10.1155/2019/3096152

**Published:** 2019-08-14

**Authors:** Lin-song Qi, Lu Yao, Xue-feng Wang, Jiu-mei Shi, Yong Liu, Teng-yun Wu, Zhi-kang Zou

**Affiliations:** ^1^Department of Aviation Physical Examination, Air Force General Hospital, 30 Fucheng Road, Beijing 100142, China; ^2^Department of Physical Examination, Cadet Bureau of PLA Air Force, 25 Minhang Road, Beijing 100195, China; ^3^Anhui Medical University, Hefei 230001, China; ^4^Department of Ophthalmology, Air Force General Hospital, 30 Fucheng Road, Beijing 100142, China

## Abstract

**Background:**

In recent decades, the prevalence rate of myopia has markedly increased, especially among teenagers. Our purpose was to determine the incidence of myopia and identify the related risk factors among schoolchildren in the experimental classes of the Air Force in China.

**Methods:**

In May 2015, this 3-year prospective cohort study enrolled 522 boys (age, 14–16 years) attending grade 10 in 16 high schools in 15 cities in China. Cycloplegic refraction was examined using retinoscopy in both eyes at the baseline and follow-up (3 years). A detailed questionnaire was completed by the students at the 3-year follow-up and included questions on parental myopia and on the total time spent doing near work and outdoor activities each week.

**Results:**

The incidence of myopia at the 3-year follow-up was 27.01% (141/522, 95% confidence interval (CI): 23.38% to 30.98%). The refractive change was −0.46 D (95% CI: −0.49 to −0.42 D). More hyperopic or less myopic baseline refraction, outdoor activity time per week ≥14 h (odds ratio (OR) = 0.464, 95% CI: 0.227 to 0.950), and reading/writing distance ≥ 30 cm (OR = 0.505, 95% CI: 0.270 to 0.944) were significant protective factors against incident myopia. Near-work time ≥28 h per week was a significant risk factor (OR = 2.579, 95% CI: 1.314 to 5.061). Parental myopia, age at the start of primary school, continuous reading/writing for ≥1 h, sleep duration per week <49 h, and one or more dietary biases were not significant risk factors (*P* > 0.05).

**Conclusion:**

A more hyperopic baseline refraction, more time spent outdoors, and longer writing/reading distance were protected against myopia onset, while more near-work time was a risk factor.

## 1. Background

Myopia is an important and widespread public health problem [1]. Indeed, the worldwide prevalence rate of myopia (defined as a spherical equivalent refraction (SER) of −0.5 D or less) is around 23% and that of high myopia is nearly 3% [2]. It has been estimated that by 2050, myopia will affect nearly half (49.8%) of the world's population, and high myopia will be found in almost a tenth (9.8%) of all people [2]. High myopia can result in cataract, glaucoma, macular degeneration, and even retinal detachment and choroidal neovascularisation, which could lead to vision loss. Myopia is highly prevalent in East Asia, particularly Japan, Singapore, China, and South Korea [3]. In China, prevalence rates of 30%–60% have been reported among children aged 15–18 years [4–7] and 80.7% among high school-aged children [4]. Myopia is an important factor that impacts the health of schoolchildren. Furthermore, in China, myopia limits the career choices of teenagers graduating from high school; for example, teenagers with myopia may not be able to become pilots or join the armed forces.

The potential causes of myopia include both hereditary and environmental factors [8]. Saw et al. [9] showed that compared to children of nonmyopic parents, children of myopic parents have a higher degree of myopia (average, 0.39 D for those with one myopic parent and 0.74 D for those with two myopic parents). Genetic factors are an important cause of myopia, especially early-onset high myopia. In contrast, school myopia has a multifactorial etiology, with environmental factors playing a major role, such as reading habits, outdoor activities, and near work [10–14]. Studies [10, 15–17] suggest that increased outdoor activity time, longer near-work distance, and decreased near-work time could reduce the incidence of myopia. However, most of these studies have focused on children in junior high school or primary school. As many students are already myopic before the age of 14, prospective studies on myopia onset in students attending senior high school are less common.

The Chinese Ministry of Education and Air Force conducts an experimental class that enrolls children aged 14–16 years who have graduated from junior high schools from all over the country, provided they pass physical examinations and academic tests. There are 16 experimental classes of the Air Force in 15 cities of China. The aim of the present study was to determine the incidence of myopia and track the progression of myopia among grade-10 to grade-12 students of the experimental class over a 3-year period in order to identify the risk factors for myopia, understand the underlying aetiological mechanisms, and formulate potential management strategies.

## 2. Methods

### 2.1. Patients and Consent

This was a 3-year-long prospective longitudinal study. All participants were recruited from the experimental classes of the Air Force. This study was approved by the ethics committee of the General Hospital of the Air Force. The study protocol complies with the tenets of the Declaration of Helsinki. We explained the objectives and methods of the study to the students and their parents and obtained both oral and written consent from both. The participants were enrolled in the study in May 2015 and were followed up for 3 years until May 2018.

The inclusion criteria were as follows: (1) uncorrected visual acuity ≥1.0 in both eyes and (2) SER between −0.25 D and +2.00 D in both eyes. The SER was calculated as the spherical power plus half of the cylindrical power. The exclusion criteria were a history of ocular surgery, ocular trauma, or an ocular disease that affected the vision.

### 2.2. Ocular Examination

All students underwent a comprehensive ocular examination including fundoscopy, slit-lamp examination, cycloplegic refraction, and 5 m distance visual acuity (Landolt C chart). The examinations were performed by two optometrists and two ophthalmologists, all of whom were trained to use standardized protocols. The pupils were dilated by instilling one drop of 0.5% tropicamide-phenylephrine ophthalmic solution (Mydrin-P, Santen, Osaka, Japan) every 5 min for 20 min in both eyes. Cycloplegic retinoscopy was performed 20 min after the administration of the last eye drops. The right eyes of the children were included in the analysis. SER ≤ −0.5 D indicated myopia, while −0.5 D < SER ≤ +2.00 D indicated nonmyopia, as children with refractions of +2.00 D or more may have other visual problems [18]. We defined incident myopia as the absence of myopia at the baseline and the development of myopia during the 3-year follow-up period.

### 2.3. Questionnaire

All participants completed a questionnaire including questions about age, age at the start of primary school, and daily activities such as near reading/writing time per week (<21 h, ≥21 h to <28 h, or ≥28 h), outdoor activity time per week (<9.33 h, ≥9.33 h to <14 h, or ≥14 h), near-work distance (≥30 cm or <30 cm), continuous reading/writing for 1 h or more (seldom/none or frequently), parental myopia (at least one parent or none), sleep duration per week (≤49 h or >49 h), and dietary bias (one/more than one or none). We defined near-reading/writing time as the total amount of time spent each week on near-work activities such as reading books, writing homework, and practicing calligraphy. We defined outdoor-activity time as the total amount of time spent each week on outdoor sports and leisure.

### 2.4. Statistical Analysis

Statistical analysis was conducted using SPSS for Windows, v24.0 (SPSS Inc., Chicago, IL, USA). We used the data of only those students who completed both the questionnaire and ocular examination. Continuous variables were expressed as mean and standard deviation or as mean (95% confidence interval (CI)). Categorical variables were compared between the myopia and nonmyopia groups by using the chi-squared test. Univariate and multivariate logistic regression analyses were used to identify the factors associated with incident myopia. Two-sided *P* values less than 0.05 were deemed statistically significant.

## 3. Results

### 3.1. Rate of Incident Myopia

**Figure 1:**
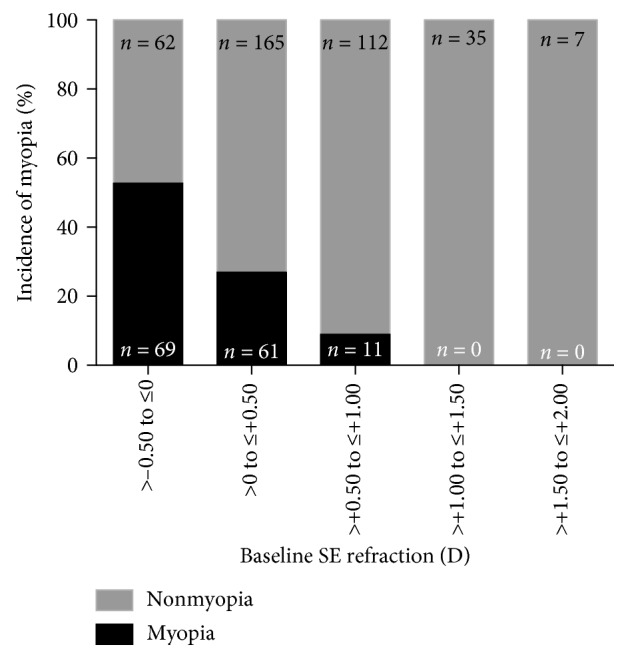
Proportion of children with and without incident myopia according to the baseline spherical equivalent refraction.

A total of 522 male students completed both the questionnaire and ocular examination. Their average age was 15.5 ± 0.6 years, and their average baseline SER (right eye) was 0.40 ± 0.46 D (Table 1). The baseline age, height, weight, and BMI did not differ between those who remained nonmyopic and those who developed myopia. By the 3-year follow-up, 141 of the 522 students had developed myopia (SER ≤ −0.5 D). Thus, the incidence of myopia was 27.01% (95% CI: 23.38% to 30.98%). The students were divided into subgroups based on their baseline SER (Figure 1). We noticed that the rate of incident myopia was greatest in the lowest two refractive categories, i.e., −0.05 D < SER ≤ 0 D (69/131 students, 52.67%, 95% CI: 44.17% to 61.02%) and 0 D < SER ≤ +0.50 D (61/226 students, 26.99%, 95% CI: 21.62% to 33.13%). The rates in the other categories were as follows: +0.50 D > SER ≤ +1.00 D, 11/123 students (8.94%, 95% CI: 5.07% to 15.31%); +1.00 D > SER ≤ +1.50 D, 0/35 students (0%, 95% CI: 0% to 9.89%); and +1.50 D > SER ≤ +2.00 D, 0/7 students (0%, 95% CI: 0% to 35.43%). The prevalence of incident myopia decreased with increasing baseline SER (*P*_trend_ < 0.0001; Figure 1).

### 3.2. Changes in Refractive Power

**Figure 2:**
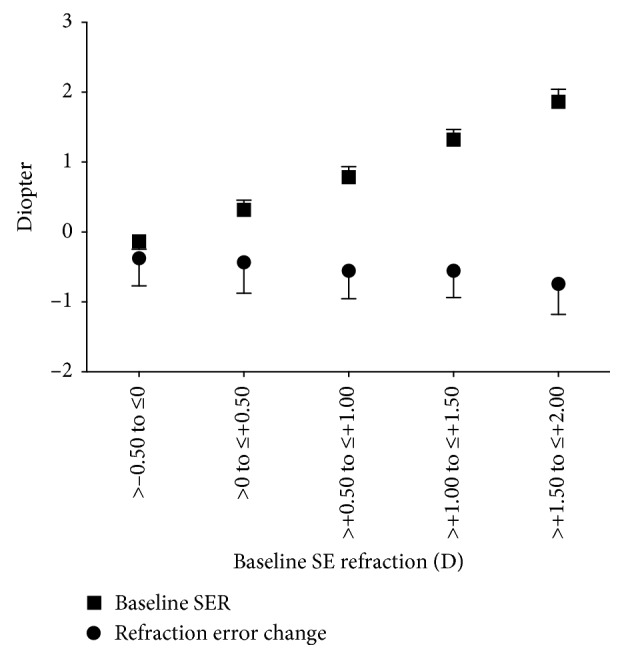
Baseline spherical equivalent refraction (SER) and change in SER from the baseline.

At the 3-year follow-up, the average SER was −0.05 ± 0.57 D. The refractive error decreased on average by −0.46 D (95% CI: −0.49 to −0.42 D, *P* < 0.0001) compared to the baseline measures. The magnitude of the SER change at the 3-year follow-up increased with increasing baseline SER as follows (Figure 2).

−0.50 D > SER ≤ 0 D: −0.37 D (95% CI: −0.44 to −0.30 D), *P* < 0.0001
2. 0 D > SER ≤ +0.50 D: −0.43 D (95% CI: −0.49 to −0.37 D), *P* < 0.0001
3. +0.50 D > SER ≤ +1.00 D: −0.55 D (95% CI: −0.63 to −0.48 D), *P* < 0.0001
4. +1.00 D > SER ≤ +1.50 D: −0.55 D (95% CI: −0.68 to −0.42 D), *P* < 0.0001
5. +1.50 D > SER ≤ +2.00 D: −0.74 D (95% CI: −1.14 to −0.33 D), *P*=0.016

### 3.3. Univariate Analysis

Univariate analyses (Table 2) revealed that the following factors were significantly associated with a decrease in incident myopia: more outdoor activity time per week (*P*=0.006, *P*_trend_=0.002), less near-work time per week (*P*=0.001, *P*_trend_=0.001), and reading/writing distance ≥ 30 cm (*P*=0.035). In contrast, having at least one myopic parent, starting primary school at ≤6 years of age, frequently reading/writing for 1 h or more, sleep duration per week ≤49 h, and one or more dietary biases were not associated with myopia onset (*P* > 0.05).

### 3.4. Multivariate Analysis

Multivariate analysis (Table 3) showed that the following factors protected against myopia onset: less myopic or more hyperopic SER at the baseline (odds ratio (OR) = 0.070, 95% CI: 0.036 to 0.137), outdoor activity time per week ≥ 14 h (OR = 0.464, 95% CI: 0.227 to 0.950), and reading/writing distance ≥ 30 cm (OR = 0.505, 95% CI: 0.270 to 0.944). In contrast, near-work time per week ≥28 h (OR = 2.579, 95% CI: 1.314 to 5.061) was associated with an increased risk for incident myopia. Parental myopia, age at the start of primary school, continuous reading/writing for 1 h or more, and sleep duration per week were not associated with myopia onset (*P* > 0.05).

## 4. Discussion

In the present study, we found that the cumulative change in the refractive index over 3 years was −0.46 D (95% CI: −0.49 to −0.42), and the proportion of children with incident myopia was 27.01%. More hyperopic or less myopic SER at the baseline, outdoor activity time per week ≥14 h, and reading/writing distance ≥30 cm were protective factors against incident myopia, while near-work time per week ≥28 h was a risk factor. Parental myopia, age at the start of primary school, and weekly sleep duration were not associated with the onset of myopia. The results of the present study were similar to those of another study by our research team [19]. In that study, Yao et al. found that more hyperopic baseline refraction was a protective factor for incident myopia, and less outdoor activity time and more near work time were risk factors for not only incident myopia but also refractive change. The students in the study by Yao et al. underwent a 20 min physical training class outdoors every day. Furthermore, that study focused on myopic shift and its risk factors at intermediate time points during a 2-year follow-up period. In the present study, we focused on detecting the general onset of myopia and its risk factors. To the best of our knowledge, the present study is the first to determine the incidence of myopia among high school-aged children in mainland China who were nonmyopic at the baseline and not given any intervention. Furthermore, our study is the first to determine the factors influencing the incidence of myopia over a 3-year follow-up period.

The SER at the baseline is the greatest individual predictor of incident myopia in schoolchildren [12, 20, 21]. French et al. [12] found that in a cohort of Australian children aged 12 years, those with a baseline refraction ≤+0.50 D were at a higher risk for developing incident myopia. A study from western China revealed that over a period of 5 years, the incidence of myopia was lower among those who were hyperopes (SER ≥ +0.50 D) at the baseline than among those who were emmetropes (−0.50 D < SER < +0.50 D) [21]. Baseline SER predicts myopia onset more accurately than even ocular measures such as axial length and corneal power [20]. Consistent with the above studies, we found that children with a more hyperopic or less myopic SER at the baseline (>+0.50 D) had a low incidence of myopia during the 3-year study period. This implies that slightly hyperopic refraction may prevent myopia onset among children aged 14–16 years, while a baseline SER ≤ +0.50 D is a potential risk factor for incident myopia.

Longer reading and writing times increase the near-work burden of the eyes. The accommodative demand increases when the eyes focus on a close target, and hence, the lens has to perform more work to ensure clarity of vision. However, the accuracy of accommodation tends to be biased because of the shortened distance, which results in accommodation lead or lag. Compared with accommodation lead, accommodation lag is more common in the development of hyperopic defocus and axial elongation. Therefore, accommodation lag has a greater impact on the incidence and development of myopia [22–24].

In our study, near-work time per week ≥28 h was obviously associated with myopia onset, which is consistent with the previous studies [16, 17, 25]. Ip et al. [17] revealed that the incidence of myopia was significantly higher in East Asian children who read 6.5 h or more per week than in Caucasian European children (32.5 h/week vs. 26.0 h/week). Furthermore, a close reading or near-work distance (<30 cm) was independently associated with incident myopia in children [17], which is consistent with our study. Wu et al. [14, 26] reported that smaller near-work distances were associated with greater myopia prevalence and greater myopic shift. Saw et al. [16] found that children in Singapore with higher myopia read nearly two more books per week than did those with lower myopia or nonmyopes. In Shanghai, China, reading/writing at close distances and 30–40 min of uninterrupted near work were found to be risk factors for myopic shift [25]. A meta-analysis showed a 2% increase in the odds of myopia onset with each additional diopter-hour per week spent doing near work [27]. These results may provide valuable information about the relationship between near work and incident myopia.

The present study showed that more outdoor activity time per week (≥9.33 h) protected against incident myopia. Numerous studies have reported that more time spent outdoors effectively prevents myopia onset and myopic shifts [15, 28, 29]. In early 1993, Parssinen and Lyyra [28] reported that myopic shifts occurred faster in schoolchildren in Finland who spent only 1.1 h per day outdoors than in schoolchildren who spent 3.2 h per day outdoors. The CLEERE group [30] found that emmetropes had longer outdoor activity hours 4 years before myopia onset and continuing till the fourth year after onset. The Sydney Myopia Study [12], which included two age cohorts of children (6 and 12 years), showed that the time spent outdoors was lower among children with incident myopia than among children who remained nonmyopic over a 5- to 6-year follow-up period. An intervention trial in Guangzhou [31] reported that one additional 40 min class of outdoor activities significantly decreased the 3-year incidence of myopia (30.4% vs. 39.5%) and change in SER (−1.42 D vs. −1.59 D). In northeast China, Jin et al. [29] found that two extra 20 min recess programs outdoors per day decreased myopia incidence by 50% over a 1-year follow-up period. Another intervention study showed that children in a suburban area in southern Taiwan who spent 80 min per day outdoors had a lower rate of myopia onset than a control group after only 1 year (8.41% vs. 17.65%) [15]. A meta-analysis found a 2% decrease in the odds of myopia for each additional hour spent outdoors per week [32]. It was also reported that children with myopic refraction had shorter outdoor activity time [33], and those who combined lower near work with higher outdoor activity had a more hyperopic refraction [34]. However, the time outdoors was not associated with progression following myopia onset [35]. The mechanism underlying the protective effect of being outdoors may be related to strong light intensity and increased dopamine release in the retina [10, 34, 36, 37].

This study has certain limitations. First, this was a 3-year observational cohort study, and there was no additional follow-up during the 3-year period. Second, the data about near work, time spent outdoors, reading/writing habits, and other related factors were obtained from questionnaires and may have been subject to recall bias. Third, the enrolled children represented a relatively homogenous group in terms of gender and age. Although the findings of our study may not be easily extrapolated to other populations, they nevertheless impart valuable information about incident myopia among high school-aged children in China who have similar academic workloads.

## 5. Conclusion

To summarize, having a more hyperopic or less myopic refraction at the baseline was an important predictor of myopia onset among high school-aged children. Additionally, spending more time on near work and less time on outdoor activities and reading/writing at a close distance were associated with an increased risk of incident myopia.

## Figures and Tables

**Table 1:** Baseline characteristics of students with and without incident myopia.

	Incident myopia	Nonmyopic	*P* value
Baseline age (years)	17.6 ± 0.6	17.6 ± 0.6	0.567
Baseline height (cm)	172.20 ± 4.63	171.58 ± 4.62	0.181
Baseline weight (Kg)	60.49 ± 7.92	59.42 ± 7.36	0.148
Baseline BMI	20.36 ± 2.37	20.16 ± 2.13	0.348

**Table 2:** Univariate analysis of potential risk factors for incident myopia.

Risk factor	Incident myopia, % (*n*)	*P* value (*χ*^2^)	*P*_trend_
Parental myopia			
0 parents	27.48 (119)	0.7525	0.1330
1 parent	23.68 (18)		
2 parents	30.77 (4)		
Outdoor activity time (per week)			
≥14 h	17.72 (14)	**0.0063**	**0.0020**
≥9.33 h to <14 h	21.66 (34)		
<9.33 h	32.52 (93)		
Near-work time (per week)			
≥28 h	30.62 (128)	**0.0010**	**0.0005**
≥21 h to <28 h	12.60 (9)		
<21 h	12.00 (4)		
Reading/writing distance			
<30 cm	42.86 (27)	**0.0065**	—
≥30 cm	24.84 (114)		
Continuous reading/writing for 1 h or more			
Seldom or none	26.64 (69)	0.8499	—
Frequently	27.38 (72)		
Age at the start of primary school			
>6 years	25.64 (40)	0.6452	—
≤6 years	27.60 (101)		
Sleep duration (per week)			
≤49 h	28.57 (90)	0.3221	—
>49 h	24.64 (51)		
Dietary bias			
One or more	30.04 (67)	0.1777	—
None	24.75 (74)		

**Table 3:** Multivariate analysis of factors associated with incident myopia.

Risk factor	OR	95% CI	*P* value
Parental myopia			
One or both	0.564	0.304–1.046	0.069
None		Reference	
Baseline SER	0.070	0.036–0.137	**<0.001**
Outdoor activity time (per week)			
≥14 h	0.464	0.227–0.950	**0.036**
≥9.33 h to <14 h	0.771	0.460–1.293	0.324
<9.33 h		Reference	
Near-work time (per week)			
≥28 h	2.579	1.314–5.061	**0.006**
<28 h		Reference	
Reading/writing distance			
≥30 cm	0.505	0.270–0.944	**0.032**
<30 cm		Reference	
Reading/writing for ≥1 h			
Frequently	0.780	0.491–1.240	0.294
None or seldom		Reference	
Age at start of primary school			
>6 years	0.855	0.527–1.388	0.526
≤6 years		Reference	
Sleep duration (per week)			
>49 h	0.968	0.600–1.562	0.895
≤49 h		Reference	

## Data Availability

The data used to support the findings of this study are available from the corresponding author upon request.

## Abbreviations

SER: Spherical equivalent refraction
OR: Odds ratio
CI: Confidence interval.
